# Parental control attitudes over their pre-school children’s diet

**DOI:** 10.1515/med-2024-1054

**Published:** 2024-09-30

**Authors:** Dulce Ivone Pinto Alves, Moniky Araújo da Cruz, Nadirlene Pereira Gomes, Amâncio António de Sousa Carvalho

**Affiliations:** Alto Ave Local Health Unit, Senhora da Oliveira Hospital, Guimarães, Portugal; Federal University of Bahia, Nursing School, Salvador, Bahia, Brazil; Health School, University of Trás-os-Montes and Alto Douro, Quinta de Prados, 5000-801 Vila Real, Portugal; External integrated member of Research Centre on Child Studies, University of Minho, Braga, Portugal

**Keywords:** child health, feeding behavior, parents, community health nursing, public health

## Abstract

It is during childhood that eating behaviors begin to form, with parents being the main agents in this process. Parents have eating habits that shape their children’s diet, both in terms of variety and quantity of food eaten. The aim is to analyze sociodemographic factors related to parental control over their children’s diet. Descriptive-correlational and cross-sectional study, with a sample of 46 parents of preschool children. An online questionnaire was used to collect data, with data processing carried out using SPSS, using descriptive and inferential statistics. The majority of respondents were mothers (89.1%), belonged to the 20–44 age group (89.1%), and were married (89.1%). The mean of the subscales of the children’s food questionnaire food restriction, pressure to eat, and monitoring was 3.266 ± 0.570, 3.109 ± 1.206, and 4.268 ± 0.848, respectively. The mean rank score for the food restriction subscale differed significantly between parents with different age groups (Mann–Whitney: *p* < 0.014), with the 45–64 age group having the highest mean rank, i.e., they restricted their children more in food. The age group is a factor related to food restriction, making it essential to take a closer look at the parents of that age group, during the health education process.

## 1 Introduction

Eating behavior is represented by all forms of interaction with food, ranging from preference for food to ingestion, representing not only what we eat, but also the elements related to the act of eating, such as the eating environment, the way and time we eat, and the reasons why people eat [1].

Researchers think that children’s eating behavior is multidetermined. Innate factors, family and school context, socioeconomic and cultural issues may be related to a child’s eating behavior. The parental practices of control, pressure, reward, and monitoring used by parents, for example, seem to influence children’s eating behavior, potentially increasing or decreasing food intake [1].

Behaviors associated with health are learned from a very early age during childhood, and are closely linked to culture. However, they can be changed as a consequence of factors associated with human development and cultural pressure [2].

Children’s eating behavior and eating habits are influenced by the family and, specifically, by their parents. In childhood, the transition to a varied diet is shaped by parents’ decisions regarding the type of feeding followed.

The practices that parents use at mealtimes to teach and reinforce children about what to eat, when to eat, and how to eat play a major role in the development of children’s eating behavior [3].

It should be noted that parents’ behavior and attitudes toward their children’s diet influence their ability to control the amount of what is ingested, the duration, and frequency of meals. Allowing the child to associate the beginning of the meal with the feeling of hunger and relating the end of the meal with the feeling of satiety, leads the child to learn to eat in moderation [4].

Parental practices regarding food, i.e., the strategies used by parents to achieve certain goals they set for their children’s diet, such as the correct way to behave at the table and what to eat, will vary according to perceptions and controlling attitudes, related to the health and development of children. These practices are influenced by the child’s characteristics, such as age, sex, eating behavior, and weight [5].

The parental food control practices that have been most studied are pressure to eat, restriction, and monitoring. Pressure to eat can be defined as a practice used to increase children’s food intake. In contrast, dietary restriction is defined as a way of limiting the intake of some foods or groups of foods. In turn, monitoring is understood as the observation carried out on the food that the child eats. Excessive use of these practices by parents can negatively or positively affect children’s intake and weight. However, some of these parental practices can influence the child to eat healthier [6].

A study carried out in Israel [7], with a sample of 174 parents of children and young people between the age of 2 and 18, aimed at evaluating the relationships between parents’ retrospective memories of their mothers’ infant feeding practices and the current form how they feed their children, highlighted the way in which adults transmit their eating practices to their children, which are strongly influenced by childhood memories, mothers’ concerns about their weight, pressure for them to eat, or restrictions on their food intake. Positive and significant correlations were also observed between parents’ current weight and concern about the child’s weight, restriction, and monitoring of food intake. The study’s most significant finding demonstrates that specific eating practices that participants mentioned retrospectively were those they currently used with their own children. Thus, this study shows how people remember mothers’ concerns about their own weight during childhood, the pressure to eat, and the restriction of food intake, as well as the transmission of these eating practices to the next generation.

Another study, carried out in Saudi Arabia [8] consisting of 209 mothers of children enrolled in pre-school education, in which the main objective was to evaluate the psychometric properties of the children’s food questionnaire (CFQ), with the study’s hypothesis being to understand whether weight perception of the child and concern about their children’s weight and diet were associated with eating practices among mothers, demonstrating that mothers who applied greater restrictions to their children monitored their children’s diet more often, and mothers who indicated that they were highly responsible for their children’s diet apply greater restriction, i.e., mothers who perceive that their child has a high weight apply greater restriction and monitoring, and mothers who are more concerned about their children’s weight will be more concerned about their diet.

The topic of eating behavior has been relevant nowadays due to the implications it has on the determination of prevalent chronic diseases such as diabetes mellitus, obesity, high blood pressure, among others [1].

In Portugal, the prevalence of chronic diseases associated with inadequate feeding is high, and is probably one of the main public health problems. No matter how efficient the health services that any society can offer its citizens, the prevention of disease and the preservation of health always depend, to a large extent, on the adoption of healthy lifestyles by people [9].

Currently, the increase in childhood obesity has reached alarming proportions and the World Health Organization (WHO) estimates that by 2025 the number of obese children on the planet will reach 75 million [10], constituting a huge public health problem throughout the world, and can be considered an epidemic [11,12].

In Portugal, according to the 2022 Childhood Report Obesity Surveillance Initiative (COSI), of the WHO/Europe COSI Portugal (2022), coordinated by the National Institute of Health Doutor Ricardo Jorge, in its capacity as WHO Collaborative Center for Childhood Nutrition and Obesity, in 2021/2022, 31.9% of children were overweight, of which 13.5% were obese. From 2019 to 2022, there was an increase of 1.6% points (11.9–13.5%) in the prevalence of childhood obesity and 2.2% points (29.7–31.9%) in the prevalence of excess weight in children [13].

In view of the above and considering the increase in the prevalence of obesity and problems related to child diet, the need for more research in this area is recognized. At governmental level, this disease has been the target of intervention and, in Portugal, despite efforts already being made in terms of treatment, such as bariatric surgery, with regard to health promotion, in school-age children and in adolescents, little attention has been paid to children in early childhood and preschool age. It is, therefore, very important to evaluate eating behavior during childhood and adolescence to understand the mechanisms that can determine adult health [14].

It is within the scope of this problem that this study is inserted, which aimed to analyze sociodemographic factors related to parental control over their children’s diet.

## 2 Methods

This is a descriptive-correlational, cross-sectional study with a quantitative approach [15]. The population was defined using the following inclusion criteria: (i) being a father/mother of children attending kindergarten, the context of this study, (ii) belong to the geographic area covered by the Community Care Unit (CCU) of Amarante, and (iii) the child is enrolled in the 2021/2022 school year.

The target population was made up of parents of pre-school children enrolled in the year 2021/2022, in kindergarten at the Basic School, the context of this study, included in the Amadeo Souza-Cardoso School Group, around 64 parents of children, attending pre-school.

The sample is defined by the exclusion criteria. The following exclusion criteria were established: (i) not having e-mail and/or digital equipment with internet access and (ii) not having answered at least 80% of the questions in the questionnaire. After applying these exclusion criteria, the sample consisted of 46 parents/guardians of preschool children, around 71.9% of the population.

The selected group was composed through the non-probabilistic, intentional, or convenience sampling technique, consisting of individuals who were available and who met the exclusion criteria.

For this study, the questionnaire was selected as the data collection instrument. We chose this data collection instrument, since it is a data collection method that allows it to be applied in the same period of time, to a large group of participants, who have the possibility of filling it out at any time they consider more appropriate, for relatively homogeneous and educated populations, being a low-cost means of data collection, fast, impersonal in nature, and can be applied online [15].

The questionnaire was created using the “Google Forms” software, which allowed more effective, quick, and practical access to study participants. This instrument included a brief presentation of the study, instructions for filling it out, and reference to the participants’ anonymity and comprised two parts: (i) the first part aimed to collect sociodemographic data and (ii) the second part included the scale called the CFQ, which assesses concern about being overweight and parents’ controlling attitudes toward their children’s diet.

This scale, called CFQ, was a method developed to assess parental practices and concerns associated with children’s diet and weight. It was initially constructed by Birch et al. [16], translated, adapted, and validated for the Portuguese population by Viana and Sinde [17], and can be applied to parents of children and young adolescents.

The CFQ consists of 31 items distributed across seven subscales/domains. Four subscales assess risk and concern about weight: “Perception of parental responsibility for their children’s diet” (3 items), “Perception of parental weight” (4 items), “Perception of child’s weight” (6 items), and “Concern about the child’s weight” (3 items). The remaining three subscales assess parental control attitudes regarding their children’s eating habits: “restriction” (8 items), “pressure to eat” (4 items), and “monitoring” (3 items). In this study, only data from these three subscales on parental eating control attitudes will be used. All items are rated on a five-point Likert scale [17]. The total scale score varies between a minimum of 28 points and a maximum of 140 points. A higher score corresponds to greater concern among parents about their children being overweight and greater control over their children’s diet.

The instrument, validated for the Portuguese population, showed good psychometric properties, with good Cronbach’s alpha values, ranging between 0.61 and 0.90, presenting an internal consistency between weak and good [18].

In order to safeguard ethical considerations, authorization was previously requested from the coordinator of the CCU of Amarante to carry out the study, as well as a request for authorization from the Amadeu Sousa Cardoso School Group, which received a favorable opinion. The UTAD Ethics Committee was also asked for an opinion on this study, which also received a favorable response (Opinion_Doc92-CE-UTAD-2021).

When data were collected, informed consent was requested from the participants, in a barrier question, with a mandatory answer, which was placed on Google Forms. Before applying the questionnaire, a meeting was scheduled with the CCU coordinator, to operationalize the data collection process. Having complied with all ethical considerations, data collection was carried out online using the Google Forms tool. As previously mentioned, the questionnaire had a brief introduction, which presented the study, as well as its objective. We began by sending the link to fill out the online questionnaire to the pre-school teachers in the school group in the context of this study, who in turn sent it to the parents who were part of the sample. The initial information made it clear that responses to the questionnaire would be anonymous, ensuring the privacy of respondents, as well as anonymity, with no cost or moral damage expected for the participant. The parents who were part of the sample completed the questionnaire at home, and the answers were available to the researcher in the Google Forms database. The data collection period ran from October 2021 to February 2022.

Statistical Package for the Social Sciences (version 27.0) database was created, where they were inserted. Subsequently, descriptive statistics and inferential statistics were used for data processing and analysis. In terms of descriptive statistics, the absolute and relative frequencies and mode frequencies were calculated for all variables, and in the case of ordinal measurement level variables, order statistics were requested and in the case of ratio measurement level variables, the calculation of measures of central tendency and dispersion (mean, median, and standard deviation) was requested. Regarding inferential statistics, parametric tests (Student’s *t* and ANOVA) were used. When the assumptions for its use were not guaranteed, alternative non-parametric tests (Mann–Whitney and Kruskal–Wallis) were used. The assumption of normality was assessed using the Shapiro Wilk test. The significance level considered was 5% [19].

## 3 Results

Of the total sample (*n* = 46), the majority of respondents were mothers (89.1%), belonged to the age group of 20–44 years (89.1%), and had married/*de facto* union status (89.1%), had basic education as educational qualifications (69.6%), and were in the employment status of an employee (69.6%) (Table 1).

**Table 1:** Characterization sociodemographic from the sample (*n* = 46)

Variables	Categories	AF	RF (%)
Degree of kinship	Father	5	10.9
	Mother	41	89.1
Age group	20–44 years	41	89.1
	45–64 years	5	10.9
Marital status	Single/separated/divorced	5	10.9
	Married/union in fact that	41	89.1
Educational level	Teaching basic	32	69.6
	Teaching higher	14	30.4
Situation labor	Employee	32	69.6
	Unemployed	14	30.4

Regarding the restriction subscale, the item that most contributed to the dietary restriction carried out by parents was item 14 “I need to make sure that my child does not eat too many sweets” in which 54.3% of parents marked the option “I agree” that they needed to make sure their child did not eat too many sweets. At the other extreme was item 19 “Do I offer my child his favorite foods in exchange for good behavior?” in which none of the parents (0%) selected the answer option “Agree,” having obtained the second highest percentage of disagreement (54.3%), as shown in Table 2.

**Table 2:** Percentage of answers to each of the CFQ items (*n* = 46), regarding the “restriction” subscale

Subscale items restriction	1	2	3	4	5
I14-Do I need to make sure that my child doesn’t eat too many sweets?	0.0	2.2	15.2	28.3	54.3
I15-Do I need to make sure that my child does not eat too many foods with a high fat content?	0.0	2.2	15.2	32.5	50.0
I16-Do I need to make sure that my child does not eat too many favorite foods?	0.0	2.2	32.6	34.8	30.4
I17-Do I purposely keep some foods out of my child’s reach?	8.7	4.3	15.2	32.6	39.1
I18-Do I offer sweets to my child as a reward for good behavior?	58.7	13.0	17.4	6.5	4.3
I19-Do I offer my child his favorite foods in exchange for good behavior?	54.3	13.0	23.9	8.7	0.0
I20-If I didn’t regulate my child’s intake, would he eat too much fast food?	32.6	17.4	23.9	10.9	15.2
I21-If I didn’t regulate my child’s intake, would he eat too many of his favorite foods?	12.0	4.3	34.8	30.4	17.4

Note: 1 – Disagree; 2 – I slightly disagree; 3 – neutral; 4 – I slightly agree; 5 – I agree.

In the pressure to eat subscale, the item that most contributed to greater agreement with this attitude was item 23 “I have to be especially careful to make sure my child eats enough,” which obtained 34.8% in the response option “I agree” and 23.9% in the “Slightly agree” option. It was also the item that had the lowest percentage in the “Disagree” response option (6.5%). On the other hand, item 24 “If my child says “I’m not hungry,” do I try to make him eat anyway?” was the one with the second lowest percentage in the answer option “Agree” 15.2% and the highest percentage in “Disagree” (26.1%) (Table 3).

**Table 3:** Percentage of answers to each of the CFQ items (*n* = 46), regarding the “pressure to eat” subscale

Pressure to eat subscale items	1	2	3	4	5
I22-Should my son always eat all the food on his plate?	26.1	13.0	26.1	21.7	13.0
I23-Do I have to take special care to make sure my child eats enough?	6.5	13.0	21.7	23.9	34.8
I24- If my child says “I’m not hungry,” do I try to make him eat anyway?	26.1	6.5	34.8	17.4	15.2
I25-If I didn’t regulate my child’s intake, would he eat much less than he should?	21.7	13.0	28.3	13.0	23.9

Note: 1 – Disagree; 2 – I slightly disagree; 3 – neutral; 4 – I slightly agree; 5 – I agree.

Finally, with regard to the monitoring subscale, the majority of parents marked the response option “Always” in all items, although item 28 “How often are you aware of foods with a high fat content that your child eats?” presented the lowest percentage. The item that most contributed to the attitude of monitoring eating habits was item 27 “How often do you pay attention to the snacks your child eats?” in which 52.2% of parents indicated the answer “Always” and 0% indicated the answer “Never.” The intake of sweets by children seemed to be the least monitored, since as a whole the “Always” and “Often” options were selected by the lowest percentage of parents (Table 4).

**Table 4:** Percentage of answers to each of the CFQ items (*n* = 46), regarding the “monitoring” subscale

Subscale items monitoring	1	2	3	4	5
I26-How often do you pay attention to the sweets your child eats?	2.2	6.5	6.5	32.6	52.2
I27-How often do you pay attention to the snacks your child eats?	0.0	6.5	6.5	34.8	52.2
I28-How often do you pay attention to the high-fat foods your child eats?	2.2	6.5	4.3	41.3	45.7

Note: 1 – Never; 2 – rarely; 3 – sometimes; 4 – often; 5 – always.

The subscale that obtained the highest average in this group was monitoring children’s diet, which obtained a value of 4.268 ± 0.818 points, with the minimum being 2.00 and the maximum 5.00 points, revealing the high level of monitoring of children’s diet children. On the opposite side was the pressure to eat subscale, which obtained an average of 3.109 ± 1.026 points, a minimum of 1.00 points and a maximum of 5.00 points, which indicates that it is the subscale that contributed least to control parental diet.

There were no significant statistical differences between the restriction score of parents with different degrees of kinship (MW: *p* = 0.972), nor with different educational levels (KW: *p* = 0.267) nor between parents with different employment status (MW: *p* = 0.829).

The restriction score differed significantly between parents with different age groups (MW: *p* = 0.014), with parents aged between 45 and 64 having a higher average rating, i.e., they restrict their children’s diet more, than parents aged between 20 and 44 years old.

No statistically significant differences were observed between the pressure to eat score of parents with different degrees of kinship (MW: *p* = 1.000), nor between parents with different age groups (MW: *p* = 0.583), nor between parents with different educational levels (KW: *p* = 0.712) nor among parents with different employment status (MW: *p* = 0.409).

There were no statistically significant differences between the monitoring scores of parents with different degrees of kinship (MW: *p* = 0.913), nor between parents of different age groups (MW: *p* = 0.650), nor between parents with different educational levels (KW: *p* = 0.690) nor among parents with different employment status (MW: *p* = 0.176).

None of the three subscales studied correlated with each other (Pearson: *p* = 0.050). However, it was possible to identify correlations with the other group of subscales. It was observed that there was a low-intensity positive correlation between the score on the perception of children’s overweight subscale and the score on the restriction subscale (Pearson: *p* = 0.033; correlation coefficient *R* = +0.316). In other words, as the score of the perception of children’s overweight subscale increases, the restriction subscale score also increases, meaning that when the perception of children’s excess weight increases, the attitude of restricting their children’s eating increases.

It was found that there is a low-intensity positive correlation between the score on the perception of children’s excess weight subscale and the score on the monitoring subscale (Pearson: *p* = 0.011; correlation coefficient *R* = +0.373), i.e., as the score for the perception of children’s overweight subscale increases, the score for the monitoring subscale increases, meaning that when the perception of children’s overweight increases, the attitude of monitoring their children’s diet increases.

It was also found that there was a low-intensity positive correlation between the score on the subscale concern about children being overweight and restriction (Pearson: *p* = 0.018; correlation coefficient *R* = +0.348), i.e., as the score on the concern about their children’s overweight subscale increases, the score on the restriction subscale increases, meaning that when parents’ concern about their children’s excess weight increases, their attitude of restricting their children’s feeding increases.

## 4 Discussion

In the sample of this study, the vast majority of parents were female, who fell into the age group of 20–44 years old, with a minimum of 20 years old and a maximum of 64 years old, and had 4 years of schooling. These results differ slightly from those obtained by the study carried out in Portugal [17], with a sample of 292 mothers, with the aim of validating the CFQ for the Portuguese population, in which all parents were female, aged between 28 and 59 years old (oldest) and completely coincides in terms of education, as the participants had between 4 and 19 years of education (from basic education to university education).

If we compare the mean values of the three subscales in our study with the mean values obtained in the study carried out in Saudi Arabia [8], with a sample of 209 mothers and their children, aiming to examine the psychometric qualities of the CFQ, in order to validate the instrument for the population of that country, it was found that the order relationship of the subscale mean values is maintained, with the highest mean being monitoring (4.44 ± 0.800) and the lowest being pressure to Eat (3.91 ± 1.140). However, in that study the mean values of the subscales were all higher than those in the present study, meaning that Saudi mothers demonstrated attitudes of greater food restriction, greater pressure to eat, and monitoring the food consumed. This difference may be due to cultural differences between the two samples and because it is a sample of older mothers.

In our study, there were only significant statistical differences between the restriction of parents with different age groups, with older parents having greater food restriction attitudes, which could be explained by the greater desire to protect their children. None of the articles consulted on this issue addressed the relationship between the CFQ subscales and the parents’ sociodemographic characteristics. Other authors report that parental practices regarding diet are influenced by the child’s characteristics such as age, sex, eating behavior, and weight, with no mention being made of the sociodemographic characteristics of the sample of parents [5].

In this sample, it was observed that there was a low-intensity positive correlation between the subscales of perception of children’s excess weight and restriction and the subscales of perception of children’s excess weight and monitoring, which is in line with the study carried out in Saudi Arabia [8], where there was a low-intensity positive correlation between the restriction subscales and the perception of children’s excess weight (*p* = 0.001; *R* = +0.20), and between the monitoring subscale and the perception of excess children’s weight (*p* = 0.01; *R* = +0.17).

With regard to the correlation between the CFQ subscales, the study that validated this instrument for the Portuguese population [17] states that the relationship between restriction and the weight concern subscale was expected, justifying that restrictive attitudes are used usually as a way of controlling weight and as a response to being overweight.

The profile of the parents who make up this sample can be considered to be a mother, aged between 20 and 44, married, with basic education, and belonging to the active population.

The strongest eating control attitude in this sample is monitoring, with pressure to eat being the least intense. The only sociodemographic factor relating to parents identified was the age group of the parents in this sample, which is related to dietary restriction, in which older parents expressed more pronounced restrictive attitudes than younger parents, constituting the factor related to that subscale.

The main limitations of this study lie in the small sample size, which limited the use of parametric tests, because the assumptions of normality and homogeneity of variances must be ensured for their use and with the fact that it is a non-random sample, which may have influenced the representativeness of the sample and affected the statistical inferences made from the sample to the population.

In terms of implications for professional practice that this study may have, knowledge of these parental dietary control attitudes can be listed, which must be taken into account when planning feeding education programs for parents, in counseling parents of children in risk of excess weight, and in the definition of weight reduction programs, by health professionals belonging to the health unit that supports the context of this study, providing health care to the parents and children of this sample. Health education sessions aimed at these parents have already been held, with the aim of training them in food control attitudes and contributing to the prevention of obesity, and pamphlets have been prepared with the main messages to reinforce this training, with a look at more attentive to older parents.
